# The high-pressure structure of (1-*x*)Na$_{0.5}$Bi$_{0.5}$TiO$_3$-*x*BaTiO$_3$ at the morphotropic phase boundary

**DOI:** 10.1038/s41598-024-69313-7

**Published:** 2024-08-13

**Authors:** Constanze Rösche, Tiziana Boffa Ballaran, Thomas Malcherek, Carsten Paulmann, Ross John Angel, Semën Gorfman, Boriana Mihailova

**Affiliations:** 1 Department of Earth System Sciences, Universität Hamburg, Grindelallee 48, 20146 Hamburg, Germany; 2 Bayerisches Geoinstitut, Universität Bayreuth, Universitätsstraße 30, 95447 Bayreuth, Germany; 3 Istituto di Geoscienze e Georisorse, CNR, Corso Stati Uniti 4, 35127 Padova, Italy; 4 Department of Materials Science and Engineering, Tel Aviv University, Wolfson Building for Mechanical Engineering, Tel Aviv, 6997801 Israel

**Keywords:** Ferroelectrics and multiferroics, X-ray diffraction

## Abstract

The pressure-induced structural changes in the perovskite-type (ABO$_3$) ferroelectric solid solution (1-*x*)Na$_{0.5}$Bi$_{0.5}$TiO$_3$-*x*BaTiO$_3$ (NBT-*x*BT) at the morphotropic phase boundary (MPB) ($x_{\text {MPB}}=0.048$) have been analyzed up to 12.3 GPa by single-crystal x-ray diffraction with synchrotron radiation. A pressure-induced phase transition takes place between 4.4 and 5.0 GPa, where the pseudocubic low-pressure phase transforms into an orthorhombic high-pressure phase with space group *Pnma*. The high-pressure phase is comprised of mixed BO$_6$ tilts and anti-polar A-cation displacements, without exhibiting coherent off-centered shifts of the B-site Ti$^{4+}$ cations that can be detected by synchrotron x-ray diffraction. Our results reveal that at ambient pressure and room temperature the NBT-$x_\mathrm{{MPB}}$BT structure possesses anti-phase BO$_6$ tilts with a relatively large correlation length and the same type of polar distortions as those present in pure NBT, but with strongly violated correlation length due to Ba$^{2+}$-induced local elastic-stress fields. For $x_{\text {MPB}}$ the effect of Ba on the mesoscopic-scale structure is compensated by a mild external pressure of only 0.7 GPa, resulting in structural features resembling those of pure NBT at ambient conditions.

## Introduction

The perovskite-type (ABO$_3$) solid solution (1-*x*)Na$_{0.5}$Bi$_{0.5}$TiO$_3$-*x*BaTiO$_3$ (NBT-*x*BT) is among the potential environmentally friendly alternatives to Pb-based ferroelectric materials. Single-crystal NBT-*x*BT exhibits a morphotropic phase boundary (MPB) at $x_\mathrm{{MPB}} \sim$0.05, across which the symmetry changes from rhombohedral to tetragonal and the material properties are enhanced^1^.

The determination of the actual symmetry at the MPB is hindered by abundant nanoscale structural heterogeneities, which have been described as polar and/or antipolar nanoregions dispersed in a matrix of different symmetry^2,3^ or as coexisting nanodomains of different symmetries^3,4^, resulting in an overall cubic-like appearance of NBT-$x_\mathrm{{MPB}}$BT^5,6^. The local structural distortions comprise off-centered shifts of A- and B-site cations^7^ as well as octahedral tilts of rhombohedral and tetragonal type^4^ ($a^-a^-a^-$ and $a^0a^0c^+$, Glazer notation^8^). Refinements to powder x-ray diffraction (XRD) data suggest that the symmetry of NBT-*x*BT ceramic samples at the MPB is *R*3*m*^9^. However, refinement parameters for *P*4*mm* were almost equally good^9^, and there is no single-crystal x-ray/neutron diffraction study confirming or refuting the proposed *R*3*m* symmetry. Therefore, the symmetry of the NBT-*x*BT structure at the MPB is still an open question.

Structural distortions that correlate only on the mesoscopic scale can be analyzed via x-ray, neutron or electron diffuse scattering (DS). At ambient conditions the DS of NBT-*x*BT changes with composition. According to XRD analyses pure NBT exhibits two types of DS: L-shaped and ellipsoidal-like/T-shaped, both along the primitive cubic (pc) $$\langle 100\rangle _{\text {pc}}$$ direction^10,11^. Considering an (*hk*0) reciprocal-lattice layer, the L-shaped DS appears around *hk*0 Bragg reflections with both *h* and *k* nonzero, whereas the ellipsoidal-like/T-shaped DS appears around reflections with *h* or *k* equal to zero, i.e., *h*00, 0*k*0. The DS extending along $$\langle 100\rangle _{\text {pc}}$$ has been interpreted as being due to the existence of subordinate platelet-like tetragonal (*P*4*bm*) regions within the monoclinic/pseudorhombohedral (*Cc*/*R*3*c*) matrix^11–13^. The asymmetry of the DS has been attributed to the different charge environments of Bi$$^{3+}$$ and Na$$^+$$^10^, which, similar to the atomic size effect in disordered alloys, transfers DS intensity from one side of a Bragg peak to another, as a result of the large difference in scattering power of the two types of atoms and low degree of chemical order^14,15^.

By increasing *x* up to $\sim$0.05^16^ the L-shaped DS changes to ellipses elongated along the $\langle 110\rangle _{\text {pc}}$ directions, implying a change in the direction of mesoscopic-scale correlated cation shifts^11,17^. The same can be deduced from the *x*-induced transformation of the DS along $\langle 100\rangle _\mathrm{{pc}}$ around *h*00 reflections into a butterfly-type with streaks along the $\langle 110\rangle _\mathrm{{pc}}$ directions^2,11,16^, indicating local-scale deviations from the average structure correlating within the $\{110\}_\mathrm{{pc}}$ planes^18^. For $x\ge 0.11$ all types of DS are strongly reduced in intensity, as the whole structure adopts symmetry *P*4*mm*^11,12^.

The Bragg-diffraction pattern of NBT-*x*BT with $x<0.11$ is further characterized by the presence of superstructure reflections, indicating that the unit cell is actually doubled. In terms of a double perovskite cell, the superstructure reflections occur at *h*, *k*, *l* all odd (*ooo*) and/or two odd and one even (*oee*) and throughout this article we will use the Bragg-peak indices in a double perovskite cell. However, for better understanding of the origin of the superstructure reflections we will consider the first Brillouin zone of a standard single perovskite cell ($Pm\bar{3}m$): the *ooo* reflections are characteristic of distortions triggered by *R*-point phonon modes, whereas the *ooe* reflections correspond to distortions driven by *M*-point phonon modes^19^. In NBT-*x*BT these superstructure reflections are associated with the co-existence of alternating anti-phase $a^{-}a^{-}a^{-}$ and in-phase $a^{0}a^{0}c^{+}$ octahedral tilts^20^ or with continuous mixed $a^{-}a^{-}c^{+}$ tilts having different coherence lengths of the out-of-phase and in-phase components^21^. For $x<x_{\text {MPB}}$ the dominant tilt pattern is anti-phase around all three fundamental crystallographic directions, while for $x>x_{\text {MPB}}$ in-phase tilting around only one direction prevails^11^.

Analyzing the crystal structure at non-ambient conditions may help to better reveal the competing local distortions and consequently, to better understand the structure at ambient pressure and room temperature. In particular, XRD analyses under high pressure are challenging, but have been successfully performed on various ferroelectric and antiferroelectric materials^10,18,22–25^. Based on the results of previous studies on Pb-based relaxors, one may expect that the application of external pressure to perovskite-type ferroelectric solid solutions may enhance the octahedral tilting and antipolar displacements of A-site cations, while suppressing the B-cation off-centred shifts and dynamical disorder^26^.

X-ray^10^ and neutron diffraction^27^ on pure NBT have shown that pressure induces a phase transition to an orthorhombic (*Pnma*) phase, which starts developing at 1.6 GPa, in coexistence with the low-pressure *R*3*c* (or *Cc*) phase. Only above $\sim$4.0 GPa does the entire structure adopt the *Pnma* symmetry, which is characterized by anti-polar order of A-cation displacements and mixed $a^{-}b^{+}a^{-}$ octahedral tilting (equivalent to $a^-a^-c^+$, but the in-phase tilting is along the **b** axis).

High-pressure Raman spectroscopy on NBT-0.048BT single crystals has revealed that between 1.0 and 4.8 GPa multi-step local-scale structural changes take place, which involve rearrangements of A-cations and the development of an octahedral tilt pattern with unequal tilts^28^. In-house XRD confirmed the occurrence of significant structural transformations above 4 GPa also on a long-range scale, but failed to resolve a symmetry lower than cubic at any pressure up to 6.1 GPa^28^.

Thus, to elucidate the MPB structure of NBT-*x*BT, here we report the results of single-crystal synchrotron XRD at pressures from ambient up to 12.3 GPa. We show that a reversible phase transition from the pseudocubic low-pressure phase to an orthorhombic *Pnma* high-pressure phase takes place at $p_c$ = 4.6(1) GPa, with orthorhombic *a* and *c* axes along $\langle 110\rangle _\mathrm{{pc}}$ and orthorhombic *b* axis $\Vert$ $[001]_\mathrm{{pc}}$. Similar to NBT, the high-pressure phase of NBT-$x_\mathrm{{MPB}}$BT consists of mixed $a^-b^+a^-$ BO$_6$ tilting and antipolar order of A-cation shifts. The pressure dependence of x-ray DS (XDS) and superstructure Bragg-diffraction peaks demonstrates that at ambient conditions the NBT-$x_\mathrm{{MPB}}$BT structure consists of the same type of polar distortions as those present in pure NBT, but their correlation length is strongly reduced by the Ba$^{2+}$-induced local elastic-stress fields, and that anti-phase $a^-a^-a^-$ BO$_6$-tilt order is the dominant tilt pattern at ambient and low pressures ($<p_c$).

## Methods

Chemically homogeneous single crystals of (1-*x*)Na$_{0.5}$Bi$_{0.5}$TiO$_3$-*x*BaTiO$_3$ with $x=0.048$ were synthesized by the top-seeded-solution-growth method^29^. The chemical composition was determined by wavelength-dispersive x-ray emission spectroscopy with uncertainty in *x* of $\pm 0.002$^5^. The sample appears pseudocubic under in-house XRD examination with $a=b=c\approx$ 3.9 Å^5,28^. For the high-pressure experiments two specimens with a size of $\sim 90 \times 95 \times 40$ and $\sim 100 \times 110 \times 40$ µm cut parallel to one of the pseudocubic $\{100\}$ planes were used for two separate runs: up to 2.3 and 7.9 GPa, respectively. The samples were loaded in Boehler-Almax diamond anvil cells^30^ (DACs) with a culet diameter of 600 µm using 200 and 390 µm thick rhenium gaskets with diameter of the drilled hole of 300 µm and a 4:1 methanol:ethanol mixture as a pressure transmitting medium . In addition, a specimen sized $\sim 40\times 40\times 30$ µm was loaded in a BX90 DAC with a culet diameter = 300 µm, using He as a pressure transmitting medium to ensure hydrostatic pressure in experiments above 10 GPa. The pressure in the sample chamber was determined using the ruby-line luminescence method^31^.

To verify the reversibility of the pressure-induced structural changes the specimen subjected to 7.9 GPa was subsequently measured in air and compared with the data collected from a pristine crystal.

Synchrotron single-crystal XRD experiments were conducted at beamline P24/EH1 at DESY using a Huber four circle Kappa diffractometer equipped with a Pilatus 1M CdTe detector. The experiments were performed with a radiation wavelength of $\lambda$ = 0.35424 Å and a sample-to-detector distance of 200 mm. For NBT-xBT the use of this high-energy beam (35 keV) ensures an absorption coefficient of $\sim$7 mm$^{-1}$ and corresponding penetration depth of $\sim$140 µm so that the entire bulk of the sample was probed. At each pressure three identical $70^{\circ }$ phi scans were collected with a step width of $0.5^{\circ }$ per frame and an exposure time of 3 s per frame, which was double-checked to be sufficient to avoid oversaturation of the strongest Bragg peaks. Intensities from the three scans were summed up in order to improve the signal-to-noise ratio and better reveal the XDS. The data were evaluated with the CrysAlisPro software package^32^. The structure of the high-pressure phase was refined using Jana2006^33^.

## Results and discussion

**Figure 1:**
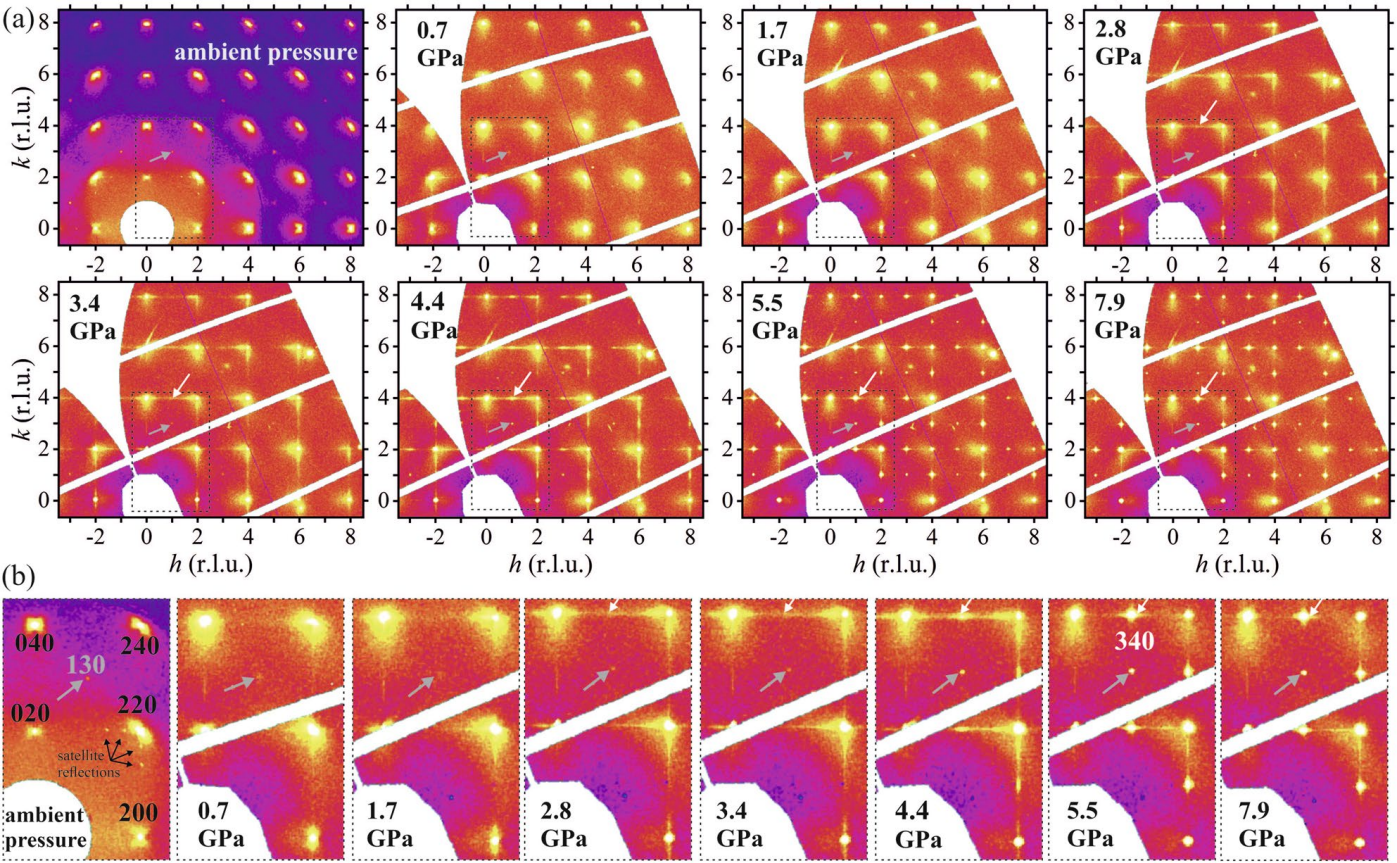
(*hk*0) reciprocal space layers of NBT-0.048BT at selected pressures from ambient pressure to 7.9 GPa. The areas marked with a dashed rectangle in (**a**) are shown enlarged in (**b**). The reflections are indexed in a doubled cubic perovskite cell. The grey and white arrows mark representative *ooe* and *oee* reflections, respectively, while the black arrows point to the satellite reflections visible around {220} reflections in the data measured at ambient pressure in air.

**Figure 2:**
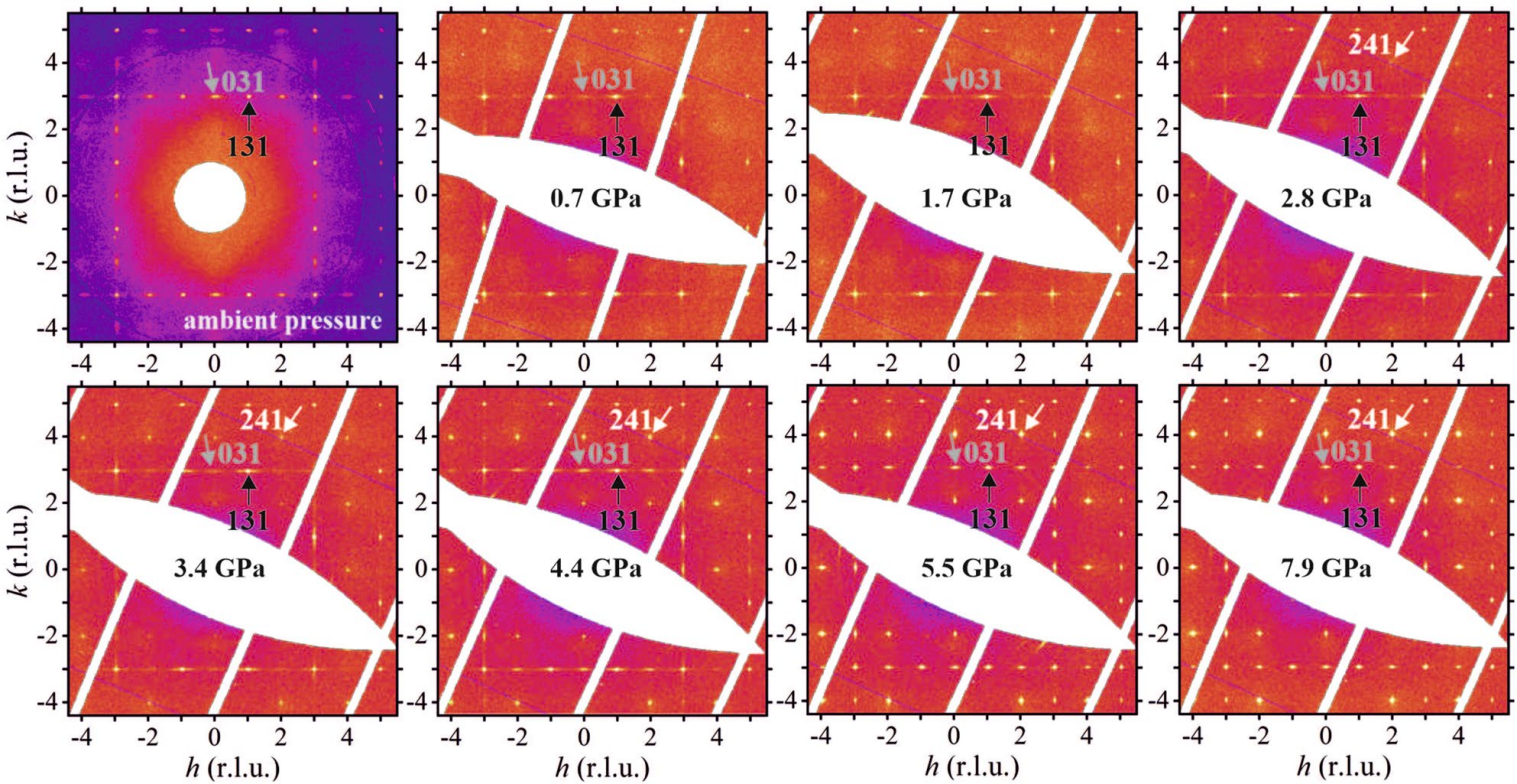
(*hk*1) reciprocal space layers of NBT-0.048BT at selected pressures from ambient pressure to 7.9 GPa. The reflections are indexed in a doubled cubic perovskite cell. The black, grey and white arrows mark representative *ooo*, *ooe* and *oee* reflections, respectively.

Due to the presence of superstructure reflections the Bragg peaks of the NBT-0.048BT crystal were indexed using a double cubic perovskite cell with the unit-cell parameter at atmospheric pressure $a_0=7.7997(4)$. Reconstructed *hk*0 and *hk*1 reciprocal-space layers of NBT-0.048BT at selected pressures up to 7.9 GPa are shown in Figs. 1 and 2. As pointed out above, the XDS scattering in the *hk*0 and *hk*1 layers is indicative of intermediate-range order of cationic off-center displacements and of octahedral tilts, respectively.

At ambient pressure the XDS around *hk*0 reflections with $h, k\ne 0$ has an ellipsoidal shape elongated along $\langle 110\rangle _\mathrm{{pc}}$ rather than an L-type shape as for pure NBT^10^, while that around *h*00 and 0*k*0 Bragg peaks exhibits a butterfly shape instead of ellipsoidal-like/T-shape along $\langle 100\rangle _\mathrm{{pc}}$. Thus, at ambient conditions XDS of NBT-0.048BT exhibits the same features as those observed for NBT-0.05BT^16^ as well as for NBT-0.056BT^2^, and resembles the XDS typical of Pb-based relaxor ferroelectrics^34^. This indicates that the presence of Ba with MPB concentrations ($x\sim 0.05$) results in nanoregions with cationic polar shifts correlated within $\{110\}_\mathrm{{pc}}$ planes^34^, which are surrounded by a pseudocubic polarizable matrix, in contrast to pure NBT, which has small platelets with correlated $\langle 100\rangle _\mathrm{{pc}}$ A-cation displacements dispersed in a rhombohedral/pseudorhombohedral matrix^10,11,35^. However, in the vicinity of {220} reflections there are satellite reflections along $\langle 100\rangle$, which both have a similar intensity and are offset by $\sim$0.3 and $\sim$0.6 reciprocal lattice units (r.l.u.). Most probably those satellite reflections are due to an incommensurate modulated structure on a length scale of approximately three times the double cubic structure ($\sim$24 Å); similar modulated structures with a different periodicity have been also observed for pure NBT and NBT-0.04BT^36,37^.

Upon pressure increase the XDS in the *hk*0 layer changes, and at moderate pressures between 0.7 and 2.8 GPa it resembles that of pure NBT at atmospheric pressure and room temperature. This shows that external hydrostatic pressure reverses the effect of chemically-induced internal local-stress fields related to the incorporation of large-size Ba$^{2+}$ at the A site^38^. The XDS of pure NBT originates from planar nanoregions with correlated $\langle 100\rangle _\mathrm{{pc}}$ shifts of A-site Na$^+$ and Bi$^{3+}$ cations within the pseudorhombohedral matrix, analogous to Guinier-Preston zones^10,37^. Apparently, moderate external hydrostatic pressure reinforces this structural state also in NBT-0.048BT, overcoming the chemical effect of embedded Ba$^{2+}$ cations. This suggests how Ba doping modifies the NBT-*x*BT structure at ambient conditions. Since the applied external pressure is isotropic, it seems unlikely to be able to change the preferred direction of polar cationic shifts within the nanodomains, while at the same time suppressing the polar long-range order in the matrix. A more plausible scenario is that at MPB concentrations barium enters both the pseudorhombohedral matrix and tetragonal nanoregions, disturbing the correlation length of coherent cationic shifts in both the pseudorhombohedral matrix and tetragonal-type platelet zones. This results in a pseudocubic matrix of uncorrelated polar distortions, which can mutually align under an externally applied electric field^37,39^ , i.e. at the MPB the matrix is polarizable, as in the case of Pb-based relaxor ferroelectrics^40–42^. Remnants of pseudorhombohedral regions, stemming from the matrix, are still preserved, which give rise to the $\langle 110\rangle _\mathrm{{pc}}$ oval-shape XDS. Due to the shrinking volume, external pressure overcomes the internal local elastic stresses in the vicinity of the A-site Ba$^{2+}$, leading to an increase in the correlation length between tetragonal-type off-center shifts as well as between local distortions of pseudorhombohedral type, which is shown by the increasing intensity and sharpness of the diffuse scattering (Figs. 1 and 2).

The satellite reflections arising from modulated structures are not observed at elevated pressures. Unfortunately it is unclear whether they are suppressed by pressure or they are just too weak to be measured inside a DAC.

At 3.4-4.4 GPa the diffuse scattering streaks in the *hk*0 layers of NBT-0.048BT have reached their maximum length and strong but still diffuse scattering appears between adjacent *ee*0 Bragg reflections (see white arrows in Fig. 1a and b), which is a precursor of new *oee* Bragg reflections that appear above 5 GPa. At 5.5 GPa the XDS is strongly reduced. However, there are still remnants of the $\langle 100\rangle _\text {pc}$ streaks, indicating that on a mesoscopic scale there is still a small fraction of tetragonal-type nanoregions that differ from the average high-pressure phase. The broad diffuse scattering at the lower-$\theta$-angle side of *eee* reflections (see e.g. 040 in Fig. 1), which results from the unequal scattering power of chemically disordered A-site Bi$^{3+}$ and Na$^{1+}$ cations^10,14,15^, is better seen at high pressures due to the suppression of the L-shaped XDS; this type of XDS persists at all pressures due to its chemical origin.

**Figure 3:**
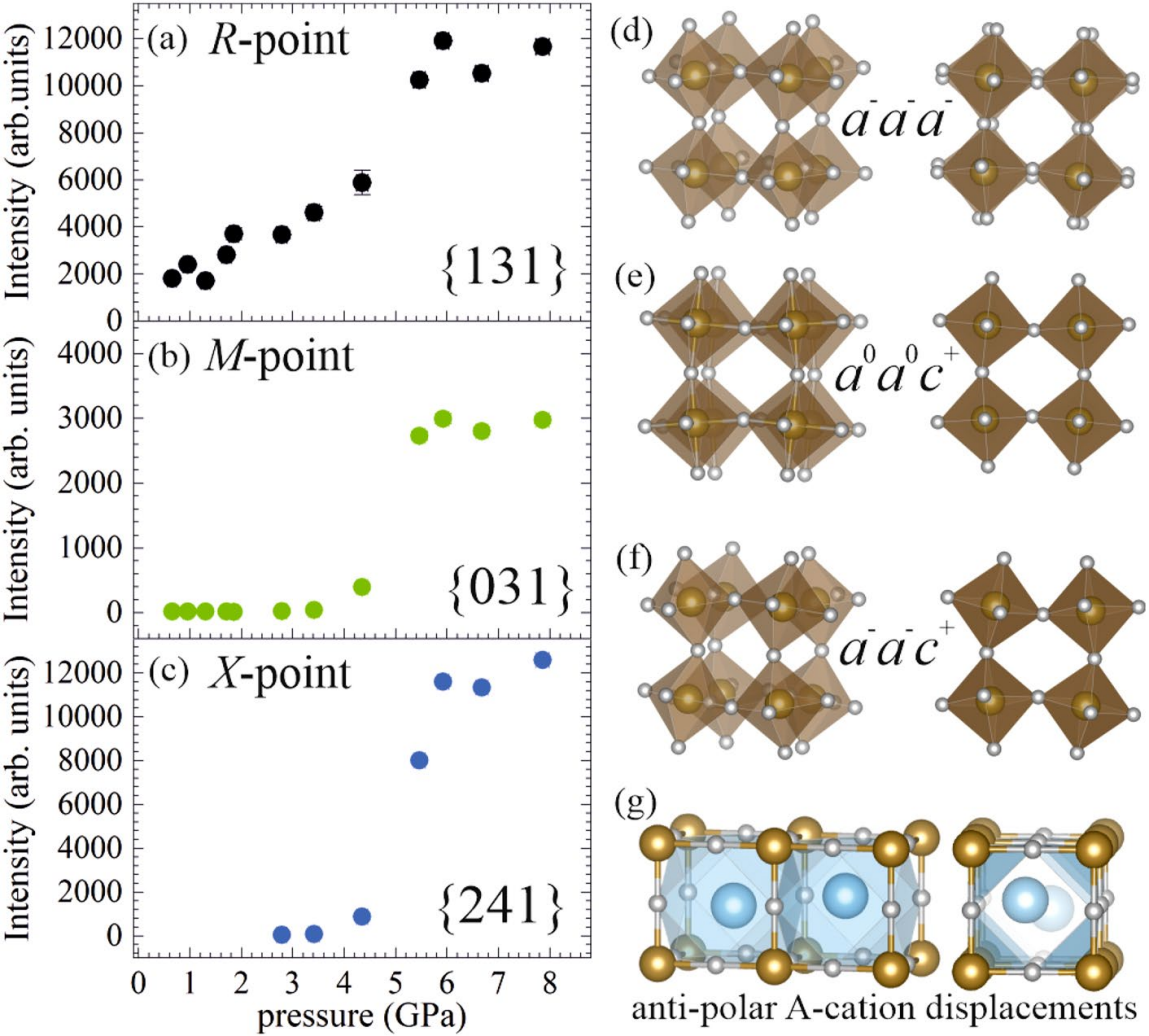
Pressure dependence of the integrated intensities of representative *ooo* (**a**), *ooe* (**b**) and *oee* (**c**) reflections. The intensities are averaged over all peaks, which are symmetry-equivalent with the Miller indices referring to the doubled pseudocubic unit cell.The octahedral tilting patterns relevant for NBT-0.048BT are shown in (**d**–**f**): pure anti-phase BO$_6$ tilting ($a^{-}a^{-}a^{-}$), in-phase tilting around one axis ($a^{0}a^{0}c^{+}$) and mixed tilting ($a^{-}a^{-}c^{+}$); right-hand-side plots are viewed along $[001]_\mathrm{{pc}}$. A sketch of anti-polar A-cation displacements is shown in (**g**); for the sake of clarity the magnitude of the displacements is exaggerated. Figures (**d**–**g**) were prepared using VESTA^45^.

The pressure dependence of the superstructure reflections gives information on the evolution of the octahedral tilt pattern. The *R*-point reflections (*ooo*) are systematically stronger than the *M*-point reflections (*ooe*); they appear clear and sharp, and increase in intensity in the whole pressure range measured (Fig. 3a). In contrast, the *M*-point reflections appear as diffuse short streaks that are elongated in $\langle 100\rangle$ directions and have a very low intensity up to 3.4 GPa, just above 3$\sigma (I)$, where $\sigma (I)$ is the estimated uncertainty of the integrated intensity. Only above 4.4 GPa does the intensity of the diffraction around the *M*-point increase strongly in intensity (Fig. 3b). These observations unambiguously confirm the dominance of the anti-phase $a^-a^-a^-$ octahedral tilt pattern (Fig. 3d) over in-phase $a^0a^0c^+$ tilting (Fig. 3e) at ambient and low pressure, which above 4 GPa evolves into a pattern of unequal BO$_{6}$ tilts, as suggested on the basis of Raman scattering analyses^28^. Precursor *oee* signals are observed already above 2.8 GPa, but they evolve into strong and sharp Bragg diffraction peaks only at 5.5 GPa (Fig. 3c). Bragg reflections of *oee* type are characteristic of distortions triggered by *X*-point phonons of the primitive cubic cell. The *oee* Bragg-diffraction maxima are significantly stronger than the *ooe* peaks, indicating that the *X*-point distortion involves A-site cationic off-centered displacements^19,43^. Hence, the co-appearance of *M*- and *X*-point reflections, along with the pre-existing *R*-point reflections, reveals a change in the octahedral tilt pattern from anti-phase to mixed (Fig. 3d,f), accompanied by the development of antipolar long-range order of A-cation shifts (Fig. 3g)^19,43^.

Even though no splitting of any of the cubic Bragg peaks is observed, the additional reflections and the disappearance of most of the diffuse scattering at 5.5 GPa clearly show that a phase transition has taken place. The set of data collected in He (at 5.0, 8.1 and 12.3 GPa) indicates that at 5.0 GPa the phase transition has already occurred, and there are no further phase transitions detected up to 12.3 GPa, as no additional peaks appear at 8.1 and 12.3 GPa (see Supplementary Fig. S3 online). However, due to smaller crystal size and different experimental conditions the integrated intensities of the He data set are not included in Fig. 3.

**Figure 4:**
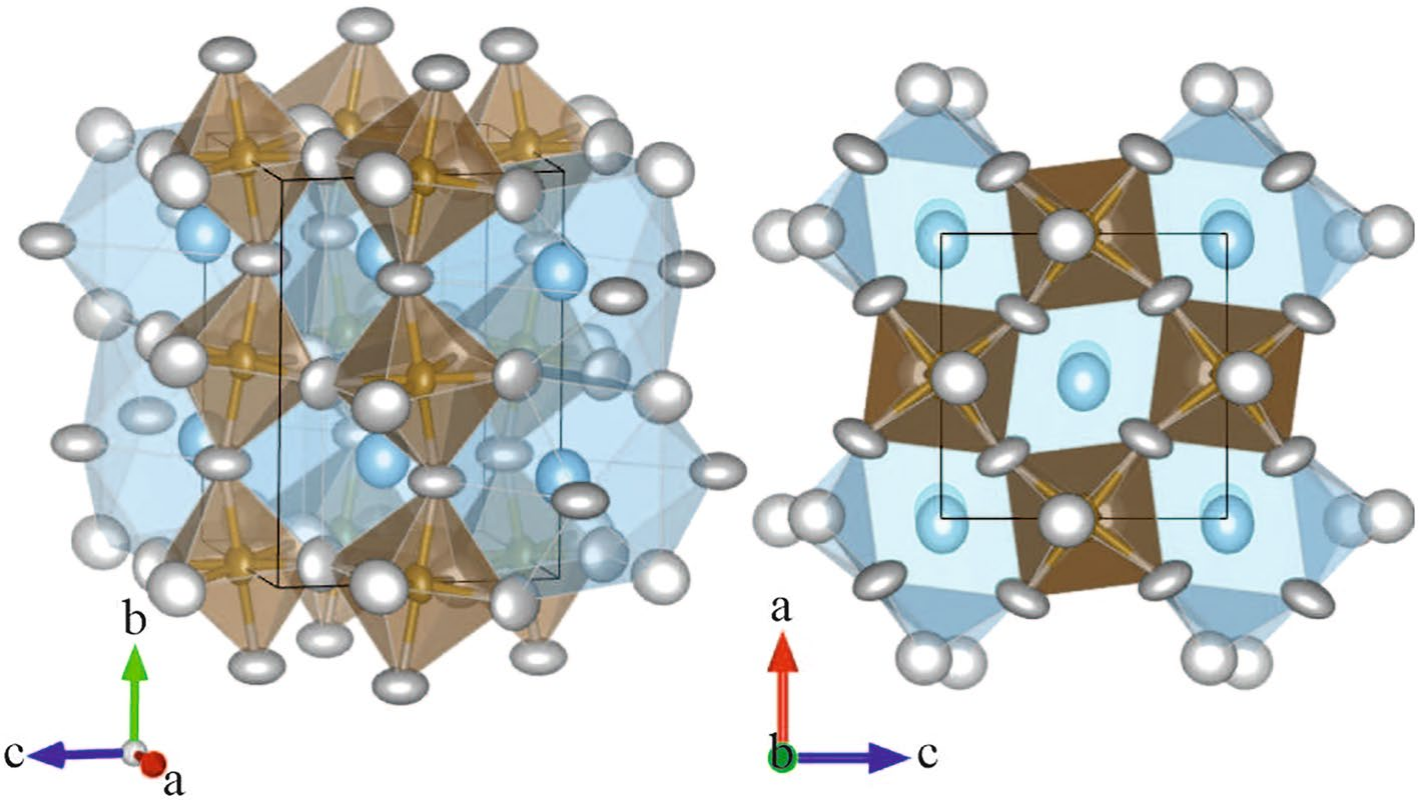
The high-pressure structure of NBT-0.048BT at 7.9 GPa refined in orthorhombic space group *Pnma*. AO$_{12}$ polyhedra are shown in blue, BO$_6$ octahedra in brown and oxygen atoms in white. All atoms are displayed as displacement ellipsoids. The corresponding refinement results are listed in Supplementary Tables S3–S5 online. This figure was prepared using VESTA^45^.

Structure refinements of the high-pressure phase are consistent with the orthorhombic space group *Pnma* (see Fig. 4), which was also found to be the symmetry to develop in pure NBT above $\sim$2 GPa^27^. The intensities used for the refinements with Jana2006^33^ were integrated with CrysAlisPro^32^ using the doubled cubic unit cell, since the orthorhombic distortion of the crystal is very small. A detailed description of the refinement details is given in the supplementary material. The unit cell and refinement parameters of NBT-0.048T at 4.4-8.1 GPa are listed in Supplementary Table S3 online. At 12.3 GPa no successful structure refinement could be achieved due to poor data quality. However, the reconstructed *hkl* layers indicate that the symmetry should still be the same (see Supplementary Fig. S3 online). The higher *R*-value ($R>5\%$) and goodness of fit (GoF = 1.26) for the refinements at 4.4 GPa show that the structure has not fully adopted the *Pnma* symmetry yet. This is also seen in the high $U_\text {eq}$ values of Bi/Na and O1 atoms, which indicate that the symmetry imposed during refinement prevents the atomic positions from being determined correctly. Also the presence of strong diffuse scattering in the reconstructed layers of the reciprocal space at 4.4 GPa (see Figs. 1 and 2) shows that the phase transition has not been completed yet. The same is seen when considering the pressure dependence of the integrated intensities (Fig. 3a–c), which shows an abrupt increase in intensity between 4.4 and 5.5 GPa. The structure refinements in *Pnma* for the data sets collected below 4.4 GPa only yielded unsatisfactory *R*-values. Hence, the low-pressure phase is pseudocubic although its structural features include octahedral tilts and A-cation polar shifts correlated at the mesoscopic scale. The unit cell parameters of the low pressure data are reported in Supplementary Table S2 online.

**Figure 5:**
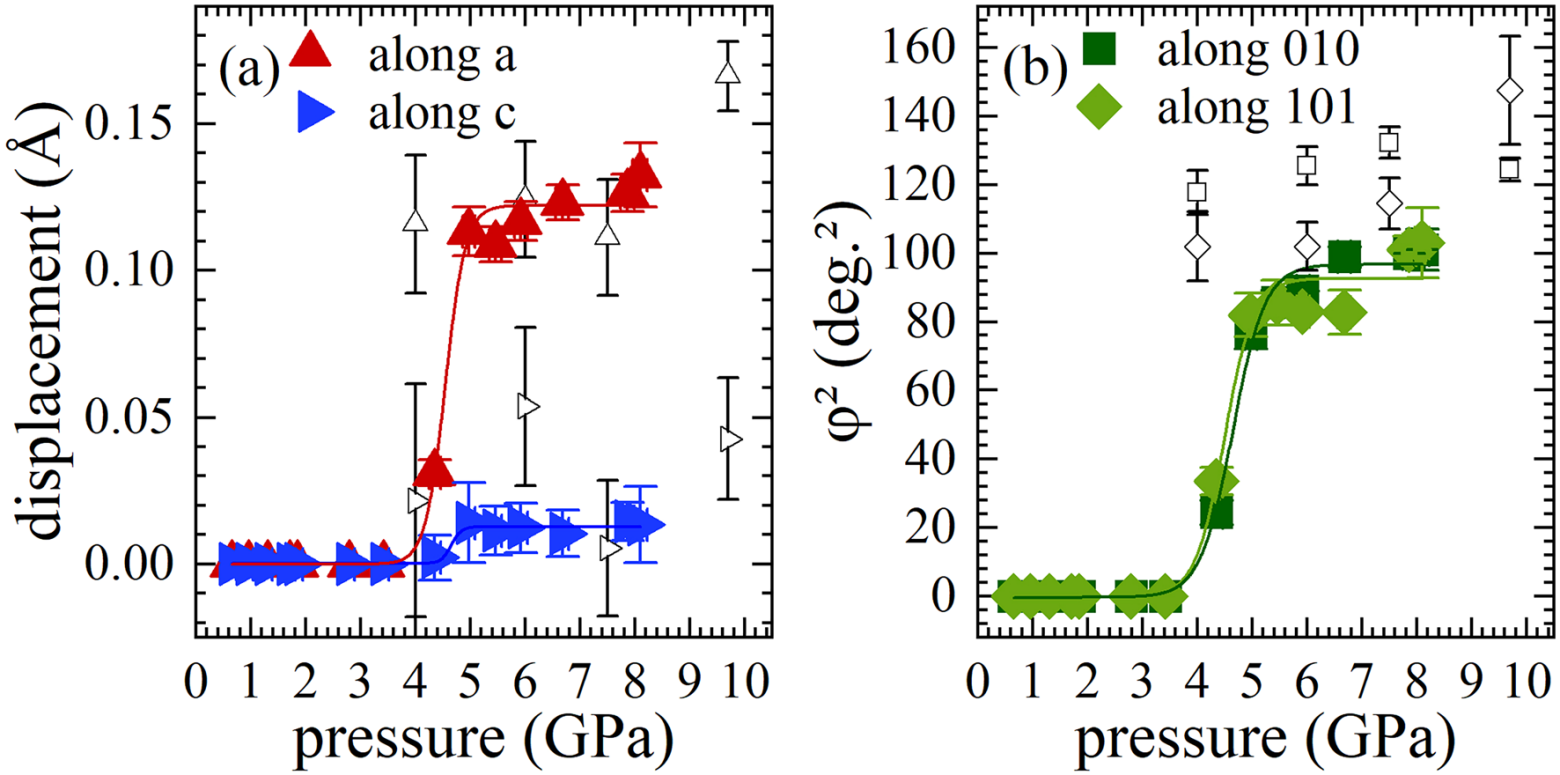
A-cation off-centering displacements $\overrightarrow {\delta }_{A}$ (**a**) and squared octahedral tilting angles $\varphi ^2$ (**b**). Filled symbols represent data on NBT-0.048BT from this study, assuming cubic structure below 4.4 GPa; lines are Boltzmann growth-function fits to the data points. Open symbols represent data derived from structure refinements on pure NBT by Thomas et al. 2005^27^.

Figure 5a shows the pressure dependence of the off-centering of the AO$_{12}$ polyhedra, calculated as the difference between the cation coordinates and the averaged oxygen coordinates $\mathbf {\overrightarrow\delta }_{A}=\overrightarrow{r}_{A}-\frac{1}{12}\times \sum _{i=1}^{12}\overrightarrow{r}_{iO}$^44^, where $\overrightarrow{r}$ refers to the corresponding position vector. In the *Pnma* phase the A-cations are only allowed to be off-set along **a** and **c** and the results show that above the phase-transition pressure the A-cation off-centering along the **a** axis significantly exceeds that along the **c** axis (Fig. 5a). The pressure dependence of the squared octahedral tilting angles $\varphi ^2 =[(180 -\angle B'-O-B'')/2]^2$ calculated from the refined atomic structures above 4 GPa is shown in Fig. 5b . A simplified model using a Boltzmann growth function to fit the $\overrightarrow {\delta }_{A}(p)$ and $\varphi ^2(p)$ data points reveals an inflection point of 4.6(1) GPa, suggesting that this is the phase transition pressure ($p_c$). However the subtle but steady increase of $\overrightarrow {\delta }_{A}$ and $\varphi ^2$ above $\sim$5.0 GPa indicates that pressure continues to enhance the tilting of BO$_6$ octahedra and off-centering of A-cations. It should be noted that in *Pnma* the Ti$^{4+}$ cations are constrained to the centre of BO$_6$ octahedra. On the other hand, the Raman scattering analysis of NBT-0.048BT indicates that the B-site off-centered shifts, although reduced, persist up to $\sim$9 GPa^28^. Therefore, the high-pressure phase of NBT-0.048BT should still comprise Ti$^{4+}$ polar displacements, which however are uncorrelated and therefore, undetectable by XRD.

On decompression the pressure-induced structural changes in NBT-0.048BT reverse, but the XRD pattern typical of the NBT-$x_\mathrm{{MPB}}$BT structural state at ambient conditions was reestablished only after a prolonged period of time (at least a few hours ). Given that the *in situ* high-pressure Raman-scattering experiments indicate that the local-scale structure recovers immediately^28^, this suggests that the dominant phase-transition mechanism is order-disorder rather than displacive, as the system needs a prolonged relaxation time to reach a thermodynamic equilibrium after the pressure release, with a structural state comprising abundant uncorrelated local ferroic distortions.

Structure data for the high-pressure phase of pure NBT at 4.0-9.7 GPa have been published by Thomas et al. 2005^27^. Within uncertainties, there is no clear difference between the calculated $\overrightarrow {\delta }_{A}$ for the high-pressure phase of NBT and NBT-0.048BT (Fig. 5a). The equivalent isotropic atomic displacement parameters ($U_{\text {eq}}$) of the Bi/Na-atoms for pure NBT are lower at all reported pressures than those we obtained from our structure refinements for NBT-0.048BT (0.019(1)-0.010(1) $\text{\AA}^{2}$ vs. 0.032(1)-0.026(1) $\text{\AA}^{2}$) (see Supplementary Table S4 online). The $U_{\text {eq}}$ parameters of Ti are in the same order of magnitude (0.006(1)-0.015(1) for $x=0$ vs. 0.012(3)-0.013(4) $\text{\AA}^{2}$ for $x=0.048$), indicating that the different A-cation $U_{\text {eq}}$ should not be due to different experimental conditions. The increased A-cation $U_{\text {eq}}$ in NBT-0.048BT can be explained by substitutional disorder induced by Ba$^{2+}$ cations, which have a larger ionic radius than Bi$^{3+}$ and Na$^+$ cations (1.61 Å vs. 1.36 and 1.39 Å). In addition, the larger $U_{\text {eq}}$ may imply that not only the fraction of off-centered A-cations is increased in the Ba-doped compound (as indicated by Raman spectroscopy^28^), but also the dynamical disorder, i.e. the amplitude of atomic vector displacements of the off-centered A-site cations is higher compared to pure NBT. The octahedral tilt angle $\varphi$ of the high-pressure phase of pure NBT^27^ tends to be higher than that of NBT-0.048BT (Fig. 5b). This indicates that the presence of large-sized Ba cations restrains the tilting of BO$_6$ octahedra not only in terms of the correlation length as at low pressure, but also in terms of the degree of tilting in the high-pressure phase.

## Conclusions

Under hydrostatic pressure, the MPB-compound NBT-0.048BT undergoes a reversible phase transition at $p_c = 4.6(1)$ GPa, with a predominantly order-disorder character. The high-pressure phase is orthorhombic *Pnma* and comprises anti-polar A-cation displacements predominantly along the *a*-axis (corresponding to the [110]$_\mathrm{{pc}}$ direction), mixed BO$_6$ tilts of type $a^-b^+a^-$, (the **b**-axis corresponding to the [001]$_\mathrm{{pc}}$ direction), and uncorrelated, reduced in magnitude, polar B-cation shifts.

The pressure evolution of the XDS below $p_c$ indicates that at MPB levels of Ba concentration, the Ba$^{2+}$ cations are embedded in both the pseudorhombohedral matrix and tetragonal-like nanoregions, which violates the correlation length of coherent A-site cations in both the matrix and nanoregions and results in a pseudocubic polarizable matrix with remnants of rhombohedral-like polar clusters. Our data indicate that for $x = x_\mathrm{{MPB}}$, the effect of chemically-induced local internal stress fields due to the incorporation of Ba$^{2+}$ cations can be overcome by a mild external hydrostatic pressure of 0.7 GPa.

The relative intensities of the superstructure diffraction scattering below $p_c$ reveals that the anti-phase $a^-a^-a^-$ BO$_6$-tilt order is the dominant tilt pattern at ambient pressure and room temperature.

### Supplementary Information

Supplementary Information 1.

Supplementary Information 2.

## Data Availability

Crystallographic Information Files (CIFs) are included as supplementary. All other datasets generated and analysed during this study are available from the corresponding author on reasonable request.
