# Supplementary material for: The high-pressure structure of (1-x)Na0.5Bi0.5TiO3-xBaTiO3 at the morphotropic phase boundary
Source: Sci Rep. 2024 Aug 13;14:18799. doi: 10.1038/s41598-024-69313-7 (PMC11322305; doi:10.1038/s41598-024-69313-7)
Supplement: Supplementary file 1 — Supplementary Information 1. [file 41598_2024_69313_MOESM1_ESM.pdf]

Supplementary Material for The high-pressure structure of $(1\text{-}x)Na_{0.5}Bi_{0.5}TiO_3\text{-}xBaTiO_3$ at the morphotropic phase boundary

Constanze Rösche[1,*], Tiziana Boffa Ballaran[2], Thomas Malcherek[1], Carsten Paulmann[1], Ross John Angel[3], Semën Gorfman[4], and Boriana Mihailova[1]

[1]Department of Earth System Sciences, Universität Hamburg, Grindelallee 48, 20146 Hamburg, Germany
[2]Bayerisches Geoinstitut, Universität Bayreuth, Universitätsstraße 30, 95447 Bayreuth, Germany
[3]Istituto di Geoscienze e Georisorse, CNR, Corso Stati Uniti 4, 35127 Padova, Italy
[4]Department of Materials Science and Engineering, Tel Aviv University, Wolfson Building for Mechanical Engineering, Tel Aviv 6997801, Israel
[*]constanze.roesche@uni-hamburg.de

Technical details of the structure refinement

The intensities used for the refinements with Jana2006[1] were integrated with CrysAlisPro[2] using the doubled cubic unit cell. The entire diffraction pattern could be indexed with a single orientation matrix. The refinements are based on the squared structure factor F^2. It was taken into account that the crystal actually consists of twin domains of lower symmetry, which all contribute with an intensity proportional to their fraction to the resulting pseudocubic diffraction pattern. Therefore 6 twin domains are included in the refinement model (see Table S1), which matches the number of possible ferroic domains due to centrosymmetrical distortion of the cubic perovskite cell along $\langle 110 \rangle$. Unit cell parameters and statistical values are reported in Table S3. The site occupation factor of Bi and Na has been fixed to 0.5 and both atoms have been restricted to have the same coordinates and anisotropic displacement parameters (ADPs). The contribution of A-site Ba has been neglected due to its small fraction in the structure (x=0.048). At 5.9-7.9 GPa the displacement parameters were refined anisotropic for all atoms. The fractional atomic coordinates and isotropic displacement parameters equivalent to the refined ADPs, U_{eq}, are reported in Table S4. At 4.4 GPa the displacement parameter of Ti had to be restrained to be isotropic in order to obtain a reasonable value. For the He data set also the oxygen atoms were refined as isotropic at 5.0 and 8.1 GPa due to poorer quality of data.

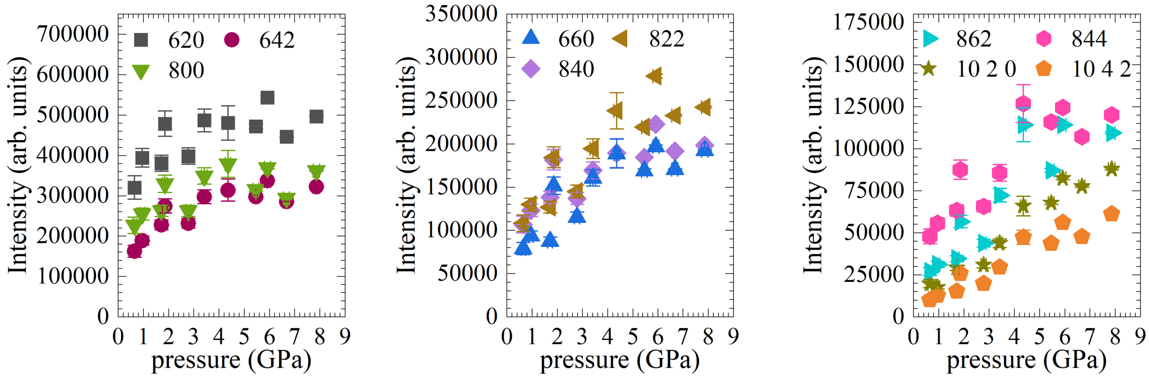

Figure S1. Pressure dependence of the integrated intensities of *eee* reflections of type $h+k+l=4n$. The intensities are averaged over all peaks, which are symmetry-equivalent with the Miller indices referring to the doubled pseudocubic unit cell.

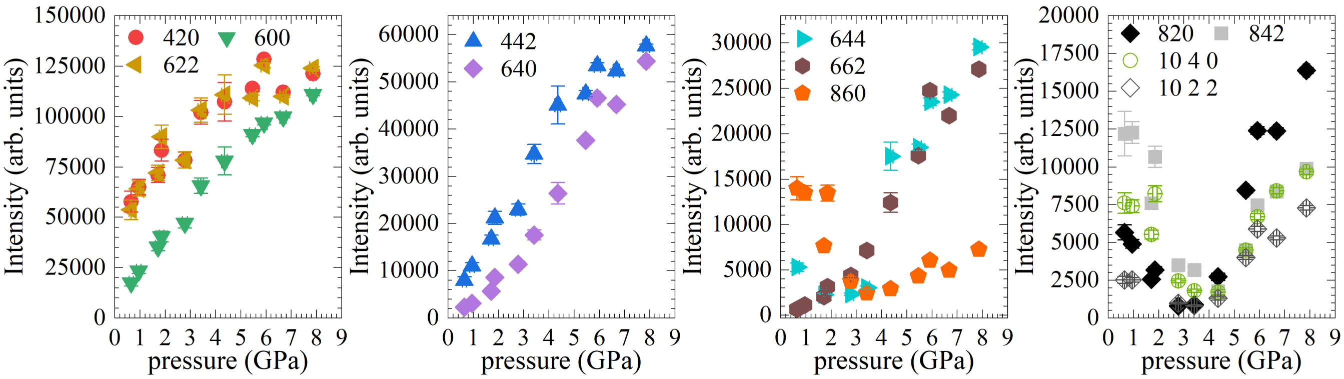

Figure S2. Pressure dependence of the integrated intensities of representative *eee* reflections of type $h+k+l = 4n+2$. The intensities are averaged over all peaks, which are symmetry-equivalent with the Miller indices referring to the doubled pseudocubic unit cell.

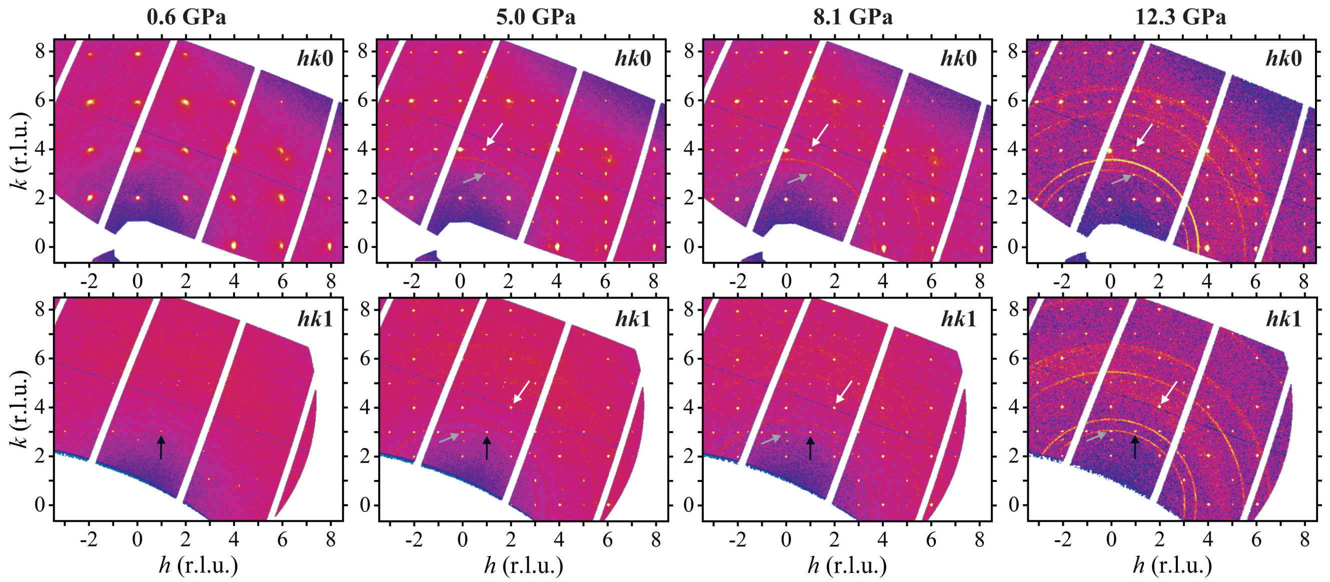

Figure S3. ($hk0$) and ($hk1$) reciprocal space layers of NBT-0.048BT from the data set measured in Helium at 0.6 to 12.3 GPa. The reflections are indexed in a doubled cubic perovskite cell. The black, grey and white arrows mark representative *ooo*, *ooe* and *oee* reflections, respectively.

Table S1. Twin domains used for the structure refinements at 4.4-8.1 GPa. The rotation axes of the twinning operations in the orthorhombic symmetry are parallel to the edge and the body diagonals of a cubic unit cell. The volume fractions of the twins composing the sample stem from the refinements in Jana2006[1]. The uncertainty for the main twin (tw1) is calculated as $\sigma(\text{tw1vol}) = \sqrt{\sigma(\text{tw2vol})^2 + \sigma(\text{tw3vol})^2 + \sigma(\text{tw4vol})^2 + \sigma(\text{tw5vol})^2 + \sigma(\text{tw6vol})^2}$.

	tw1	tw2	tw3	tw4	tw5	tw6
		2-fold rotation around $(\bar{1},0,1)$ $(\|\|[100]_c)$	3-fold rotation around $[\bar{2},1,0]$ $(\|\|[\bar{1}1\bar{1}]_c)$	3-fold rotation around $[2,1,0]$ $(\|\|[111]_c)$	3-fold rotation around $[0,1,2]$ $(\|\|[\bar{1}11]_c)$	3-fold rotation around $[\bar{2},\bar{1},0]$ $(\|\|[\bar{1}\bar{1}\bar{1}]_c)$
Pressure (GPa)			fraction			
4.35(11)	0.148(19)	0.146(9)	0.177(9)	0.178(8)	0.167(9)	0.184(9)
4.97(12)	0.199(17)	0.169(11)	0.199(7)	0.257(7)	0.027(6)	0.149(5)
5.46(12)	0.168(10)	0.147(4)	0.134(4)	0.106(3)	0.218(5)	0.227(5)
5.92(12)	0.170(9)	0.152(4)	0.227(5)	0.230(5)	0.115(3)	0.105(3)
6.67(13)	0.239(8)	0.242(5)	0.098(3)	0.112(3)	0.165(4)	0.144(3)
7.86(13)	0.166(9)	0.153(4)	0.112(3)	0.102(3)	0.235(5)	0.232(5)
8.09(13)	0.181(18)	0.193(12)	0.250(7)	0.211(7)	0.144(5)	0.021(6)

Table S2. Unit cell parameters of the doubled pseudocubic perovskite unit cell of the low pressure phase of NBT-0.048BT up to 3.4 GPa derived from profile fitting with CrysAlisPro[2].

Pressure	*a* (Å)
0.00010(1) GPa	7.7997(4)
0.65(9) GPa	7.7712(8)
0.95(9) GPa	7.7620(7)
1.30(9) GPa	7.7530(6)
1.71(10) GPa	7.7424(10)
1.85(10) GPa	7.7415(8)
2.79(11) GPa	7.7184(8)
3.41(11) GPa	7.7052(7)

Table S3. Results of the structure refinements in space group *Pnma* of the single-crystal XRD data of NBT-0.048BT above 4.4 GPa. The parameter b stems from the unit cell refinement with CrysAlisPro[2] using a doubled pseudocubic perovskite cell, while a and c stem from the transformation of the cubic to an orthorhombic unit cell with $a = c = b/\sqrt{2}$ and uncertainties $\Delta a = \Delta c = \Delta b/\sqrt{2}$.

Pressure	4.4 GPa	5.0 GPa	5.5 GPa	5.9 GPa	6.7 GPa	7.9 GPa	8.1 GPa
Unit cell parameters (Å)	$a = 5.4309(4)$ $b = 7.6804(5)$ $c = 5.4309(4)$	$a = 5.4163(3)$ $b = 7.6598(4)$, $a = 5.4163(3)$	$a = 5.4159(2)$, $b = 7.6592(3)$, $c = 5.4159(2)$	$a = 5.4092(2)$, $b = 7.6498(3)$, $c = 5.4092(2)$	$a = 5.3964(2)$, $b = 7.6316(3)$ $c = 5.3964(2)$	$a = 5.3855(2)$ $b = 7.6163(3)$ $c = 5.3855(2)$	$a = 5.3818(3)$, $b = 7.6110(4)$, $a = 5.3818(3)$
R (obs/all)	0.0579/0.0630	0.0471/0.0584	0.0437/0.0448	0.0427/0.0440	0.0407/0.0423	0.0355/0.0364	0.0522/0.0652
GoF (obs/all)	1.26/1.05	1.13/1.00	1.09/1.05	1.08/1.02	1.06/1.00	1.04/0.99	1.16/0.99
Number of reflections (obs/all)	239/362	226/351	406/460	404/460	392/447	399/445	217/351
Variables refined	28	20	33	33	33	33	20

Table S4. Fractional atomic coordinates *x*, *y* and *z* and equivalent isotropic displacement parameters U_{eq} (Å^2) from structure refinements in *Pnma*.

4.4 GPa	*x*	*y*	*z*	U_{eq}[1]
Bi/Na	-0.5054(1)	0.75	0.5004(4)	0.050(1)
Ti	0.5	0.5	0	0.016(1)
O1	-0.7574(8)	-0.0191(15)	0.2377(5)	0.057(3)
O2	-0.5011(5)	0.25	1.036(2)	0.023(3)

5.0 GPa	*x*	*y*	*z*	U_{eq}[2]
Bi/Na	-0.5189(2)	0.75	0.4981(3)	0.031(1)
Ti	0.5	0.5	0	0.014(1)
O1	-0.7781(13)	-0.0266(9)	0.2226(13)	0.021(2)
O2	-0.5061(14)	0.25	1.056(2)	0.025(2)

5.5 GPa	*x*	*y*	*z*	U_{eq}[3]
Bi/Na	-0.5176(2)	0.75	0.4979(2)	0.032(1)
Ti	0.5	0.5	0	0.013(2)
O1	-0.7787(9)	-0.0286(8)	0.2208(10)	0.022(2)
O2	-0.5076(10)	0.25	1.057(2)	0.024(3)

5.9 GPa	*x*	*y*	*z*	U_{eq}[3]
Bi/Na	-0.5192(2)	0.75	0.4977(2)	0.029(1)
Ti	0.5	0.5	0	0.013(3)
O1	-0.7786(11)	-0.0289(7)	0.219(1)	0.021(2)
O2	-0.5073(10)	0.25	1.056(2)	0.021(3)

6.7 GPa	*x*	*y*	*z*	U_{eq}[3]
Bi/Na	-0.5201(2)	0.75	0.4981(2)	0.027(1)
Ti	0.5	0.5	0	0.012(3)
O1	-0.7814(9)	-0.0311(6)	0.2197(9)	0.018(2)
O2	-0.5082(10)	0.25	1.056(2)	0.020(3)

7.9 GPa	*x*	*y*	*z*	U_{eq}[3]
Bi/Na	-0.5209(2)	0.75	0.4973(2)	0.026(1)
Ti	0.5	0.5	0	0.013(4)
O1	-0.7817(10)	-0.0293(5)	0.216(1)	0.020(2)
O2	-0.5077(10)	0.25	1.0623(10)	0.020(2)

8.1 GPa	*x*	*y*	*z*	U_{eq}[2]
Bi/Na	-0.5222(2)	0.75	0.4975(3)	0.023(1)
Ti	0.5	0.5	0	0.009(1)
O1	-0.7838(18)	-0.0289(12)	0.2167(17)	0.017(2)
O2	-0.5073(19)	0.25	1.063(3)	0.025(3)

[1]data set measured in methanol:ethanol 4:1; refinement: displacement parameters Bi/Na and O anisotropic, Ti isotropic
[2]data set measured in Helium; refinement: displacement parameters Bi/Na anisotropic, Ti and O isotropic
[3]data set measured in methanol:ethanol 4:1; refinement: displacement parameters of all atoms anisotropic

Table S5. Anisotropic displacement parameters from structure refinements in *Pnma* at 4.4-8.1 GPa.

4.4 GPa	U_{11}	U_{22}	U_{33}	U_{12}	U_{13}	U_{23}
Bi/Na	0.0566(14)	0.0581(16)	0.0338(11)	0	0.00044(12)	0
O1	0.073(6)	0.066(5)	0.031(3)	0.0114(17)	0.034(3)	0.0047(13)
O2	0.032(5)	0.010(4)	0.025(5)	0	0.0025(11)	0
**5.0 GPa**	U_{11}	U_{22}	U_{33}	U_{12}	U_{13}	U_{23}
Bi/Na	0.0279(9)	0.0360(13)	0.0303(13)	0	0.0001(3)	0
**5.5 GPa**	U_{11}	U_{22}	U_{33}	U_{12}	U_{13}	U_{23}
Bi/Na	0.0328(8)	0.0359(7)	0.0277(12)	0	−0.00003(14)	0
Ti	0.007(4)	0.012(4)	0.022(4)	0.0009(2)	0.0003(4)	0.0008(4)
O1	0.017(3)	0.0256(16)	0.025(3)	−0.0036(18)	0.0121(10)	−0.0032(17)
O2	0.025(6)	0.016(4)	0.029(5)	0	−0.0017(16)	0
**5.9 GPa**	U_{11}	U_{22}	U_{33}	U_{12}	U_{13}	U_{23}
Bi/Na	0.0310(8)	0.0321(6)	0.0241(11)	0	0.00013(14)	0
Ti	0.009(5)	0.015(4)	0.014(5)	0.0006(2)	−0.0004(6)	0.0011(4)
O1	0.013(3)	0.0239(17)	0.027(3)	−0.004(2)	0.0074(13)	−0.004(2)
O2	0.018(6)	0.009(3)	0.035(5)	0	0.0002(16)	0
**6.7 GPa**	U_{11}	U_{22}	U_{33}	U_{12}	U_{13}	U_{23}
Bi/Na	0.0278(8)	0.0277(6)	0.0240(11)	0	0.00036(15)	0
Ti	0.012(6)	0.015(3)	0.010(6)	0.0005(3)	0.0004(5)	0.0013(4)
O1	0.011(2)	0.0193(15)	0.025(3)	−0.0057(17)	0.0075(11)	−0.0008(18)
O2	0.012(6)	0.009(3)	0.037(6)	0	−0.0020(16)	0
**7.9 GPa**	U_{11}	U_{22}	U_{33}	U_{12}	U_{13}	U_{23}
Bi/Na	0.0281(8)	0.0261(6)	0.0225(11)	0	0.00023(11)	0
Ti	0.020(8)	0.013(4)	0.007(6)	0.0003(3)	−0.0004(4)	0.0006(3)
O1	0.014(2)	0.0232(17)	0.024(3)	−0.0042(17)	0.0069(10)	−0.0006(18)
O2	0.024(6)	0.010(3)	0.026(4)	0	−0.0003(15)	0
**8.1 GPa**	U_{11}	U_{22}	U_{33}	U_{12}	U_{13}	U_{23}
Bi/Na	0.0202(9)	0.0244(12)	0.0237(13)	0	0.0005(3)	0

References

1. Petříček, V., Dušek, M. & Palatinus, L. Crystallographic computing system JANA2006: General features. *Zeitschrift für Kristallographie - Cryst. Mater.* **229**, 345–352, DOI: 10.1515/zkri-2014-1737 (2014).

2. Agilent. *CrysAlis PRO*, Agilent Technologies Ltd, Yarnton, Oxfordshire, England (2014).
